# The effect of household heads training on long-lasting insecticide-treated bed nets utilization: a cluster randomized controlled trial in Ethiopia

**DOI:** 10.1186/1475-2875-11-99

**Published:** 2012-03-30

**Authors:** Amare Deribew, Zewdie Birhanu, Lelisa Sena, Tariku Dejene, Ayalu A Reda, Morankar Sudhakar, Fessehaye Alemseged, Fasil Tessema, Ahmed Zeynudin, Sibhatu Biadgilign, Kebede Deribe

**Affiliations:** 1 Department of Epidemiology, Jimma University, Jimma, Ethiopia; 2 Department of Health Education and Behavioral Sciences, Jimma University, Jimma, Ethiopia; 3 Department of Public Health, College of Health Sciences, Haramaya University, Harar, Ethiopia; 4 Department of Medical Laboratory and Pathology, Jimma University, Jimma, Ethiopia; 5 Faculty of public Health, Jimma University, Jimma, Ethiopia

**Keywords:** Malaria, LLITN, Effectiveness, Training on LLITN, Ethiopia

## Abstract

**Background:**

Long-lasting insecticide-treated bed nets (LLITN) have demonstrated significant impact in reducing malaria-related childhood morbidity and mortality. However, utilization of LLITN by under-five children is not satisfactory in many sub-Saharan African countries due to behavioural barriers. Previous studies had focused on the coverage and ownership of LLITN. The effect of skill-based training for household heads on LLITN utilization had not yet been investigated. A cluster-randomized trial on the effect of training of household heads on the use of LLITN was done in Ethiopia to fill this knowledge gap.

**Methods:**

The study included 22 (11 intervention and 11 control) villages in southwest Ethiopia. The intervention consisted of tailored training of household heads about the proper use of LLITN and community network system. All households in each group received free LLITN. Data were collected at baseline, six and 12 months of the follow up periods. Utilization of LLITN in the control and intervention villages was compared at baseline and follow up periods.

**Results:**

A total of 21,673; 14,735 and 13,758 individuals were included at baseline, sixth and twelfth months of the project period. At the baseline survey, 47.9% of individuals in the intervention villages and 68.4% in the control villages reported that they had utilized LLITN the night before the survey. At the six month, 81.0% of individuals in the intervention villages and 79.3% in the control villages had utilized LLITN. The utilization of LLITN in all age groups in the intervention villages was increased by 17.7 percentage point (95% CI 9.7-25.6) at sixth month and by 31.0 percentage point (95% CI 16.9-45.1) at the twelfth month. Among under-five children, the LLITN utilization increased by 31.6 percentage point (95% CI 17.3-45.8) at the sixth month and 38.4 percentage point (95% CI 12.1-64.7) at the twelfth months of the project period.

**Conclusion:**

Household level skill-based training has demonstrated a marked positive effect in the utilization of LLITN. The effect of the intervention steadily increased overtime. Therefore, distribution of LLITN should be accompanied by a skill-based training of household heads to improve its utilization.

**Trail registration:**

Australian New Zealand Clinical Trials Registry (ACTR number: ACTRN12610000035022).

## Background

Long-lasting insecticide-treated bed nets (LLITN) have demonstrated a significant impact in the reduction of malaria-related childhood morbidity and mortality [1-5]. However, the utilization of LLITN is greatly affected by behavioral factors in countries south of the Sahara. Previous studies had given much emphasis on the coverage and ownership of LLITN [5-7]. Nonetheless, all owned LLITN are not being used practically. A study conducted in the two biggest regions of Ethiopia showed that 91% of households owned at least one LLITN but only 65% of the LLITN had been utilized the night before the survey [6]. A more recent study in Ethiopia has documented a significant reduction of LLITN utilization [7], which indicates the need for sustainable mechanism of improving LLITN utilization.

In Africa, the utilization of LLITN is affected by practical and technical difficulties related to fixing of the net above the mat and the design of the house [8,9]. Previous studies have also documented that people tend to use LLITN during the rainy season and peak malaria transmission periods [10]. To resolve the technical difficulties and improve LLITN utilization, the community should have access to information and skill-based trainings. Previous studies have not addressed behavioural aspect of LLITN utilization [6-8]. As far as could be ascertained, no other study has addressed the impact of household heads training on the proper utilization of LLITN. In this study, the hypothesis was that empowering household heads on the proper use of LLITN through training will increase its utilization by all age groups including under-five children.

## Methods

### Study area

The study was conducted in a malaria endemic area in Gilgel Gibe Field Research Centre (GGFRC). The GGFRC, with a total population of 50,000, was established in 2005 and is located 260 Km south-west of Addis Ababa, the capital city of Ethiopia [9,11]. The study area lies between latitudes 7°42'50"N and 07°53'50"N and between longitudes 37°11'22"E and 37°20'36"E, at an altitude of 1,734-1,864 metres above sea level. It has annual rainfall of between 1,300 and 1,800 mm with a mean annual temperature of 19°C. The main socio-economic activities of the local communities are mixed farming involving the cultivation of staple crops and husbandry [11]. According to previous studies, the prevalence of malaria in the area among under five children ranged from 8.3 to 10.5% [9,11]. A possible ecological change due to the construction of a hydroelectric dam might have contributed for the high prevalence of malaria in the area [9,11].

### Study design and sampling

The study utilized a cluster-randomized trial. The primary endpoint of the trial was differences in utilization of LLITN among under-five children between the control and intervention groups. Secondary endpoints included fever and malaria prevalence among under-five children. The study population consisted of heads of households, under-five children and pregnant women in twenty-two *Gots *(villages). Heads of the households were selected to be trained on the proper use of LLITN. The major rationale of selecting heads of the households is that they are the decision makers in all matters (including health issues) within a household. To avoid contamination, villages in the field research centre were stratified into North and South of the reservoir of the dam. *Gots *in the north direction of the reservoir were selected randomly as intervention whereas those villages in the south direction of the dam were selected to be control groups and both groups are within the same distance (10-15 Km) from the reservoir. A total of 22 *Gots *(11 intervention and 11 control villages) were included as mentioned in detail elsewhere [9].

### The intervention and data collection procedures

The intervention and data collection procedure is published elsewhere [9]. In brief, the study had three phases: preparatory, intervention and evaluation phases. Baseline survey was undertaken from February to March, 2009. After the baseline survey, the intervention phase was started in May 2009 and continued for one year. The intervention consists of tailored training of household heads on the proper use of LLITN and establishing community network system. Nine village residents were identified and given training of trainers (TOT) about the proper utilization of LLITN for five days. The village residents gave training to all heads of the households in each *Got *using posters and manuals prepared in local language. After a five-day training, demonstration on use of LLITN was undertaken in the rural *tukul *(huts commonly built out of mud and wood walls, and hay roofs) houses in the study villages. Community network system was also established in the intervention villages to make the intervention sustainable. The network system consisted of regular communication among district health office, trained village residents and the researchers [9]. A malaria focal person at the district health office, village residents and the researchers had regular monthly meeting to discuss utilization of LLITN and challenges, additional need of LLITN and anti-malarial drugs. The focal person at the district health office was responsible to monitor and supervise the activities of the trained village residents. The investigators had also regular independent supervision at randomly selected villages. The village residents report their activities to the malaria control programme and investigators regularly. The investigators, the malaria control team, the trained village residents and the Health Extension Workers (HEW) have regular monthly monitoring meeting to discuss progress of activities and challenges. Health extension workers are female health workers who completed high school and trained for one year to give minimum health packages to the community at the health post and household level.

During the baseline survey, seventy-seven percent of households had at least one LLITN. After the training, 12,500 LLITN were distributed to the control and intervention *Gots*. LLITN were obtained from UNICEF Ethiopia and households were given LLITN based on family size. All households in both intervention and control groups were given at least two LLITNs, however for families with large size (≥ 8), three LLITN were provided. In total 4,135 households (2,105 interventions and 2,030 controls) were included in the distribution. As of May 2009, each household in the intervention villages has been visited by the trained village residents monthly to check for the proper use of LLITN and the occurrences of malaria.

The proper use of LLITN was evaluated using observation and checklist. The checklist consisted of age and sex of each household member and questions related to proper utilization of LLITN. The proper hanging of the net in the four angles of the bed/mattress using locally made wooden materials, status of the net (new or old), and the proper placement of the net under the mattress or bed were checked in each visit. The intervention and control villages were monitored using the same checklist to monitor the utilization of LLITN. The trained village residents also collect information on the occurrence of malaria/fever using questionnaire. Mass blood investigation was made at baseline, 6 and 12 months of the project to assess malaria and anaemia.

A total of eight (four among women and four among men) focus group discussions (FGD) were conducted in the control and intervention villages at the baseline and 12^th ^months of the project period. The FGD participants were selected purposively in consultation with the *Kebele *(small administrative units) leaders. Individuals who were supposed to have adequate information were included in the FGD. The FGD were conducted by experienced investigators using a check-list and all the FGD were tape recorded.

### Data management and statistical analysis

Data were entered into computer, edited, cleaned, and analysed using SPSS-16 and STATA version 10. Both unadjusted and adjusted effect sizes for the trial endpoints are presented. The adjusted results were presented by taking into account potential confounders such as age and sex. Indirect standardized proportion ratios were compared between the intervention and control villages at 6- and 12-months of the project period by taking into account baseline cluster size, sex and age. The indirect standardized figures, as well as baseline proportions were then compared using a *t*-test. Multivariate analysis was conducted using complex surveys logistic regression in STATA. A *p*-value of less than or equal to 0.05 was considered to be significant for all tests. The presentation has followed the CONSORT statement guidelines for cluster randomized trials [12]. Analysis of the qualitative data was done manually. All focus group discussions' (FGD) notes and tap records were translated from Afan Oromo (local language) to English. The translated texts were read by the investigators several times and mutually exclusive and meaningful categories/themes were developed. The data were then coded according to the category system. Data belonging to each category were retrieved, assembled and viewed to get meaningful interpretation based on the objectives of the study. The interpreted qualitative data were presented together with the quantitative results to triangulate the findings. Individuals' quotes were used to illustrate important findings.

### Ethical considerations

The proposal was approved by Jimma University and the WHO ethical committee. Written consent was obtained from caretakers of under-five children.

## Results

### Baseline characteristics

A total of 21,673; 14,735, and 13,758 individuals in the 22 villages were included at baseline sixth and twelfth months of the project periods (Table 1). The utilization of LLITN was assessed among 4,034; 2,967, and 2,796 under-five children at baseline, sixth and 12^th ^months respectively. At the baseline survey, perceived utilization of LLITN by all age groups in the control villages (68.4%) was higher than the intervention villages (47.9%) (*p *= 0.001). Similarly, utilization of LLITN by under-five children in the control villages (76%) was higher than the intervention villages (52.1%) (*p *= 0.000). The detailed baseline result was published elsewhere [9]. After the baseline assessment, more than 12,500 LLITN were distributed for all villages equally and its utilization was then assessed by the trained village residents.

**Table 1:** Baseline characteristics of participants and outcome variables

Variable		Baseline		6 months		12 months	
		
		Control, n (%)	Intervention, n (%)	Control, n (%)	Intervention, n (%)	Control, n (%)	Intervention, n (%)
Age in months							

5		1670 (18.0)	2364 (19.1)	1288 (19.0)	1679 (21.1)	1299 (18.9)	1497 (21.7)

5-14		2767 (29.9)	3683 (29.8)	2312 (34.1)	2609 (32.8)	2360 (34.4)	2085 (30.2)

≥ 15		4824 (52.1)	6321 (51.1)	3175 (46.9)	3672 (46.1)	3199 (46.6)	3326 (48.1)

	Total	9261	12368	6775	7960	6858	6908

Sex							

Male		4703 (50.8)	6184 (50.0)	3418 (50.5)	3876 (48.7)	3437 (50.1)	3360 (48.7)

Female		4562 (49.2)	6192 (50.0)	3357 (49.5)	4084 (51.3)	3427 (49.9)	3534 (51.3)

	Total	9265	12376	6775	7960	6864	6894

LLITN utilization by all age group the previous night							

Yes		6338 (68.4)	5928 (47.9)	5365 (79.3)	6448 (81.0)	3718 (71.7)	4577 (97.1)

No		2929 (31.6)	6450 (52.1)	1397 (20.7)	1511 (19.0)	1471 (28.3)	135 (2.9)

	Total	9267	12378	6762	7959	5189	4712

LLITN utilization by under-five children the previous night							

Yes		1269 (76.0)	1232 (52.1)	978 (76.2)	1536 (91.5)	624 (61.2)	1195 (97.6)

No		401 (24.0)	1132 (47.9)	306 (23.8)	143 (8.5)	396 (38.8)	29 (2.4)

	Total	1670	2364	1284	1679	1020	1224

### Six months outcomes

The LLITN utilization rate in all age groups has increased in both arms (79.3% in the control villages versus 81.0% in the intervention villages) (Table 1). This gave an effect size (mean difference) of 17.7 percentage-points (95% CI 9.7-25.6, *p *= 0.000). The utilization of LLITN increased among under-five children in both arms at the sixth month (76.2% in the control villages vs. 91.5% in the intervention villages). Among children under five, an effect size of 31.6 percentage-points (95% CI 17.3-45.8, *p *= 0.001) was observed (Table 2).

**Table 2:** Utilization of LLITN in the control and intervention villages in south-west Ethiopia

Variable	Baseline		6 months		12 months		Effect size (difference of differences)^£^			
							
							At 6 month		At 12 months	
	
	**Control**, n (%)	**Intervention**, n (%)	**Control**, n (%)	**Intervention**, n (%)	**Control**, n (%)	**Intervention**, n (%)	^**¥**^**Uadj percentage;** Adj (95% CI)	*P*-value	**Uadj percentage**, Adj (95% CI)	*P*-value
LLITN utilization by all age groups the previous night										

Yes	6338 (68.4)	5928 (47.9)	5365 (79.3)	6448 (81.0)	3718 (71.7)	4577 (97.1)	*22.2%;* 17.7 (9.7, 25.6)	0.000	*45.9%*; 31.0 (16.9,45.1)	0.000

No	2929 (31.6)	6450 (52.1)	1397 (20.7)	1511 (19.0)	1471 (28.3)	135 (2.9)				

Total	9267	12378	6762	7959	5189	4712				

LLITN utilization by under-five children the previous night										

Yes	1269 (76.0)	1232 (52.1)	978 (76.2)	1536 (91.5)	624 (61.2)	1195 (97.6)	*39.2%;* 31.6 (17.3, 45.8)	0.001	*60.3%;* 38.4 (12.1, 64.7)	0.009

No	401 (24.0)	1132 (47.9)	306 (23.8)	143 (8.5)	396 (38.8)	29 (2.4)				

Total	1670	2364	1284	1679	1020	1224				

Had fever in the last two weeks										

Yes	709 (17.4)	700 (8.3)	1590 (23.5)	534 (6.7)	858 (12.5)	165 (2.4)	-*7.7%*; -15.0 (-20.8, - 9.3)	0.000	-*1.0;*, -2.1 (-5.6, 1.4)	0.224

No	3369 (82.6)	7773 (91.7)	5174 (76.5)	7405 (93.3)	5998 (87.5)	6744 (97.6)				

Total	4078	8473	6764	7939	6856	6909				

Sought treatment within 24 hours of onset of fever										

Yes	388 (55.1)	419 (60.5)	485 (30.8)	152 (30.5)	128 (14.9)	27 (16.9)	-*5.7%;* -2.2 (-17.2, 12.7)	0.763	-*3.4;*, -6.7 (-23.7, 10.2)	0.419

No	316 (44.9)	273 (39.5)	1088 (69.2)	346 (69.5)	729 (85.1)	133 (83.1)				

Total	705	692	1574	498	857	160				

^¥^Uadj = unadjusted effect size, adj = adjusted effect size for age, clustering and sex, CI = Confidence interval, ^£^Difference of difference at 6 month = (follow up at six months minus baseline) intervention- (follow up at six months minus baseline) control, ^£^Difference of difference at 12 month = (follow up at 12 months minus baseline) intervention- (follow up at12 months minus baseline) control. Negative value shows reduction of the respective outcome measures. LLITN = Long-lasting insecticide-treated bed nets

### Twelve months outcomes

The LLITN utilization rate in all age groups had increased from 81.0% at the sixth month to 97.1% at the twelfth month in the intervention villages and it had decreased from 79.3% to 71.7% in the control villages. This gave a mean difference of 31.0 percentage-points (95% CI 16.9-45.1, *p *= 0.000). The LLITN utilization rate among under-five children was increased from 91.5% at the sixth month to 97.6% at the 12^th ^months of the follow up period. The effect size of the LLITN utilization rate in under-five children was 38.4 percentage-points (95% CI 12.1-64.7, *p *= 0.009) (Table 2).

### Community perception on the utilization of LLITN and burden of malaria

The community in the intervention villages perceived that malaria has declined as a result of the distribution of the free LLITN and the continuous training given to them.

*"In our village, the burden of malaria is declining since free bed nets are given to us and we are using them properly based on the training given to us"*, a woman at one of the intervention village.

There was a difference in the skill and knowledge of the FGD participants concerning the use of bed nets at the baseline and 12^th ^month of the project period. At baseline, most participants did not know how to hang the nets properly. On the contrary, at the 12^th ^month, most FGD participants in the intervention villages clearly described how to properly use the bed nets. A 50 years-old farmer at the intervention villages confidently described the proper use of LLITN as follows:

"We dig four holes in the four directions of our bed and put four strong sticks in the holes. We then stretched the bed net over the bed/mattress and tie its four angles on the sticks. We always make sure that the edges of the bed nets are properly put under the mattress so as to prevent the entry of mosquitoes during the night."

In the intervention villages, priority concerning utilization of LLITN was given for *Sebiyi *(pre-school children) and pregnant women. However, some FGD participants mentioned that some households gave priority for the head of the household and wives.

On the other hand, all the FGD participants in the control villages described that the burden of malaria is still very high particularly among children and women. Lack of knowledge and skill to use LLITN, inability of the insecticide spay to kill the mosquitoes and ever increasing of the population of mosquito in the area were the three major reasons for the high burden of malaria in the control villages. Proper use of LLITN in the control villages is greatly affected by lack of training of the community and health education materials such as posters. As a result of lack of skill and knowledge, most people use the LLITN for other purposes such as bed sheets and cloaks for bags or other properties. A male farmer participant in the control villages angrily described the burden of malaria and lack of proper utilization of LLITN as follows:

"I have never witnessed a decline of malaria in our village. It is affecting and killing our children every time. Most people in our villages do not know how to use the bed nets given to them."

## Discussion

This study investigated the effect of training of households on the LLITN utilization. In the presence of serious challenges to sustain LLITN utilization, such types studies are important to guide programme planners and policy makers. Unlike previous studies, which focused on LLITN ownership, this study combined ownership and skills oriented training to empower the community to maximize LLITN utilization. In light of declining trend of LLITN utilization in Ethiopia, the experience of this study may help decision makers to adopt similar community empowering strategies particularly for people residing in malaria risk areas.

The main finding of this study indicates that training of household heads has significant impact on LLITN utilization. Specifically, training of households heads on LLITN use increased the utilization of LLITN use by 17.7 percentage-points in the immediate term in high malaria transmission season and the effect was sustained and pronounced (31.0 percentage-points) in low malaria transmission season. This significant increase occurred despite equivalent distribution of LLITN in both the intervention and control villages. This reaffirms previous reports that ownership doesn't guarantee utilization [13].

The utilization of LLITN in the three surveys on the project was much higher than reports from elsewhere in the country [7,14,15]. The continuous demographic surveillance system and the health education messages by the Gilgel Gibe Field Research centre staffs and the Jimma University graduate programme students might have contributed for the high utilization of LLITN compared to other sites. However, the intervention *Gots *are much better in utilization of LLITN compared to the control groups. Malaria is prevalent in the area due to the possible ecological disruption as a result of the construction of the hydroelectric dam [11]. The community members also witnessed the high burden of malaria in all the seasons particularly in the control villages. The burden of malaria and anaemia in the control and intervention villages is presented elsewhere [16].

LLITN utilization in both intervention and control villages increased at the six months survey. This period is rainy and of high malaria transmission season where previous reports indicated more frequent use of nets [10]. However, it is interesting that the intervention had significant impact on utilization at the sixth months of the project. Importantly, the impact of the intervention is more pronounced among under-five children with an effect size of 31.6 and 38.4 percentage-points at the sixth and 12^th ^months of the project period. This may imply that the training has also brought change in prioritizing vulnerable groups. Nonetheless, one should realize that such priority might come because of distribution of multiple LLITN per household. In any case, the findings of this study indicate that intervention had significant impact on utilization of LLITN utilization by under-five children.

During the twelve month survey, which is dry, hot and of low malaria transmission season, the utilization has decreased from 79.3% to 71.7% in the control groups and increased in the intervention villages from 81.0% to 97.1%. One encouraging observation here is that the intervention increased utilization over extended period with different weather conditions and malaria transmission seasons. The decline in the control villages may further prove seasonal variation of LLITN use [10]. Such consistent improved utilization of LLITN after a year were not observed in other interventions intended to increase utilization. For instance, studies looking at the effect of incentives on changing health behaviours have found that incentives are effective in the short-term, but often fail to influence long-term behavior [13,17]. This highlights the importance of skilled-based education which has long-lasting effect on changing behaviour and sustaining the desired outcomes.

After LLITN were owned by households, ensuring utilization has been a challenge. Different methods have been tried to address major barriers through individual level health education and mass communications [18,19]. The underlying assumption in such initiatives is that providing information about the risks associated with malaria and the benefits of LLITN will lead to higher perceived value of LLITN and thus increased use of LLITN. Such approaches, however, may not be the most efficient means of ensuring LLITN utilization. Incentives have also been tried for increasing LLITN utilization. Nonetheless, incentive induced utilization was shown to last for short period [12]. Studies have documented the low utilization rates in sub-Saharan Africa are related to technical difficulties [8,9]. Therefore, interventions focusing on skill-based training such as the one employed in this study are very important. The qualitative findings also confirmed that the intervention was beneficial to ensure proper utilization of LLITN and identify priority groups such as under-five children and pregnant women. The community acceptance of the intervention and acceptance with local authorities implies the applicability of the intervention in other settings. Shortly after completion of the research, local health authorities implemented the intervention in the control and other surrounding villages.

The government of Ethiopia has deployed 30,578 health extension workers (HEWs) serving almost all villages in rural areas [20,21]. The HEWs are widely involved in diagnosis and treatment of malaria and health education in the community [21,22]. The skilled-based training of household heads described in this study can be linked to the activities of the HEW to make it sustainable.

This study has utilized a rigorous study design to assess the impact of skill based training on the utilization of LLITN in programme settings. However, the study has some limitations. Firstly, self-reported use of LLITN might lead to over reporting. Second, the sample size in the 6^th ^and 12^th ^months is lower than the baseline and this might introduce information bias. The long-term sustainability and the cost-effectiveness of such interventions is not clearly known. Lastly, information bias might be introduced during recording and reporting of data regarding LLITN utilization and presence of fever.

## Conclusion

Community empowerment through training of household heads on the use of long lasting insecticide treated nets (LLITN) was found to be an effective intervention in increasing the utilization of LLITN in both low and high transmission seasons. The effect of the intervention was more visible among under-five children. Adoption of similar LITTN use mobilization strategies is recommended in high risks areas, such as water development projects. Integrating such skill-based training in the existing health care setting would make it sustainable. Further research is suggested to evaluate the long-term impact of the intervention and its cost-effectives.

## Competing interests

The authors declare that they have no competing interests.

## Authors' contributions

AD conceived the study and was involved in the design, coordination, field supervision, analysis and drafted the manuscript. AAR, TD and FT were involved in the data analysis and reviewed the article. LS and FA participated in the design, field supervision and report writing. ZB and MS were involved in field supervision and writing of the qualitative report. AZ was involved in the laboratory quality control and field supervision. SB and KD involved in organizing the data, interpreting the findings and drafted and reviewed the article. All authors read and approved the manuscript.
